# Emotional regulation and Arnold’s self-ideal: a way to flourishment

**DOI:** 10.3389/fpsyg.2024.1425850

**Published:** 2024-08-14

**Authors:** Fátima Ruiz-Fuster, Aurora Bernal-Martínez de Soria, Martín F. Echavarría

**Affiliations:** ^1^Universidad Internacional de la Rioja, Logroño, Spain; ^2^Department of Theory and Methods in Educational and Psychological Research, Universidad de Navarra, Pamplona, Spain; ^3^Department of Psychology, Universitat Abat Oliba CEU, CEU Universities, Barcelona, Spain

**Keywords:** emotional regulation, flourishing, self-ideal, Arnold, appraisal, theory of emotions, motivation

## Abstract

The convergence of researchers in the fields of flourishing, moral psychology, and social–emotional studies has reached a stage where developing a theory that connects emotional regulation and flourishing is meaningful. This theoretical investigation aims to uncover insights from the research of Magda B. Arnold, renowned for her theory of emotions, and lesser-known for her notion of the self-ideal, regarding the relationship between emotional regulation and flourishing. Our initial hypothesis posits that Arnold’s concept of self-ideal provides a framework for understanding how to foster emotional regulation in individuals by directing it toward constructive life objectives. To achieve this, we explore the current state of emotional regulation and flourishing and the relationship between these concepts; we consider the interconnectedness of emotion and self-ideal within Arnold’s theory and analyze its potential to serve as a foundation for building a theory relating flourishing and emotional regulation. We find in Arnold’s theory substantial ideas about the relationship between emotional regulation, flourishing, and self-ideal, as well as emerging empirical research relating to these themes. We conclude that Arnold’s research can serve as a catalyst for developing psychological intervention models that enhance emotional regulation and promote a flourishing life.

## 1 Introduction

Emotional regulation and flourishing are two growing fields in the realms of psychology and education. However, the interplay between emotional regulation and flourishing is a less explored topic, albeit no less relevant. The aim of this research is to study this subject. The procedure involved reviewing the research conducted by Magda B. Arnold, known for her theory of emotions, to find clues about the relationship between emotional regulation and flourishing. Our starting hypothesis is that Arnold’s notion of self-ideal allows us to understand how promoting emotional regulation in individuals serves as a means for them to achieve constructive life goals. As we will see later, such goals are an important element of flourishing as it is currently conceived (Vittersø, 2016a). This proposal is particularly innovative due to the limited research linking emotional regulation with flourishing, and because existing studies focus on showing the correlation between specific emotional regulation strategies and flourishing (Barber et al., 2010; Richard-Sephton et al., 2024). However, the proposed approach is novel in its aim to theoretically link emotional regulation and flourishing as a preliminary step before examining the connection between specific emotional regulation strategies and flourishing.

Most of the research on Arnold has focused on her contribution to the psychology of emotion (Gasper and Bramesfeld, 2006; Kappas, 2006; Reisenzein, 2006; Shields, 2006). However, recently, the literature has focused on investigating other aspects of her theory: her inspiration from Thomistic psychology (Echavarría, 2020; Pugen, 2020), the anthropological foundations of her personality theory (García-Alandete, 2024), as well as the value of her theory of emotions in the current framework of flourishing (Valenzuela, 2024).

Of particular value to this work is considering how Hulsey and Hampson (2014) link Arnold’s concept of self-ideal to moral identity. These authors assert, drawing on Arnold’s theory, that acting in accordance with this self-ideal leads to harmony and integration of the personality, optimizing emotional response. This assertion is reiterated by Cornelius (2006): the goal in the organization and integration of the personality, in Arnold’s theory, is the coherence between emotional responses and the person’s highest ideals, citing Arnold and Gasson (1954), p. 306:

If… emotion is to be instrumental in self-actualization, the objects of emotion must be harmonized with the person’s larger goal as a human being. If these objects are seen in their real value, if they are seen in the proper perspective of man’s final end, then the judgment that they are suitable will be objective and well ordered.

This last statement is directly related to the topics of flourishing and personal purpose, subjects of study that have grown in interest in current psychology. It also shows the link between these concepts and emotional regulation: emotions need to be regulated to achieve constructive life goals, which will allow the integration of the personality, as mentioned by Arnold (1969a,b, 1970).

Valenzuela (2024), on the other hand, directly links Arnold’s theory of emotions with the current concept of flourishing. According to this author, the relationship between emotions and Arnold’s self-ideal is related to flourishing understood as eudaimonia. Specifically, she shows that Arnold’s concept of happiness goes beyond a hedonistic conception of pleasure, relating it to the meaning of life and to a fundamentally eudaemonic content. Likewise, she suggests that the self-ideal not only leads to maturity, but also to the flourishing of the personality. Thus, the positive emotions that, according to Arnold, allow us to pursue the self-ideal and, especially, the desire for happiness, more than other emotions, would help us to pursue goals that are valuable and meaningful for our lives.

Building on this recent publication on Arnold (Valenzuela, 2024), we will propose the interrelation between the concepts of emotional regulation and flourishing based on Arnold’s model and her concept of self-ideal. Before that, we will present the state of the art on the theory of emotional regulation and flourishing, focusing on the possible relationship between both processes and conceptions. The study we present aims to respond primarily to the following question: How can Arnold’s psychological theory contribute to a better understanding of emotional regulation, flourishing, and the relationship between them? To accomplish this, we must first investigate the following issues: Does flourishing manifest in emotional regulation? Do current theories on flourishing include any reference to emotions and their regulation? Is flourishing an objective or could it be an objective of emotional regulation?

## 2 Emotional regulation

Emotional regulation is a concept that has been widely studied in recent decades and is a field characterized by definitional chaos (Gross, 1999). In Table 1, we include a summary of the theories discussed in this section and their main focus. According to Petrova and Gross (2023), in the year 2022, more than 30,000 articles were published on this topic. In their article, these authors reflect on the future of research on emotional regulation, focusing on interpersonal emotional regulation, on the tactics for carrying out emotional regulation strategies, and on the temporal plane of this process. These three keys set the roadmap for the next generation of emotional regulation researchers. However, in order to understand the current state of the question, it is necessary to refer to the most relevant theories that have dominated during the last decades. In this regard, Gross (1999, 2015), a renowned author in this field, stands out, formulating one of the most relevant theories in this field at the end of the 20th century. He distinguishes between the process of emotional generation and the process of emotional regulation (Gross et al., 2011) and presents a process model of emotion generation, based on different researchers among which we find Arnold, Lazarus, Frijda and others. This model acknowledges the concept of valuation or appraisal initiated by Arnold (1969a, 1970), understanding cognition not only as strictly intellectual or rational, but also as sensory knowledge (Echavarría, 2020), reflected in this author’s intuitive appraisal (Kappas, 2006).

**Table 1:** Emotional regulation (ER).

The **process** of emotional regulation	Thompson (1991)	ER refers to the extrinsic processes that **monitor, evaluate and modify emotional reaction**s to accomplish one’s goals.	
	Gross (1999)	ER refers to the processes that **exert an influence on the emotions** we have, when we have them, and on how we experience and express them. *Situation-attention-appraisal-response*	The set of responses can be modulated with different strategies-adapative (reappraisal) or non adaptative (suppresion)
	Gross et al. (2011)	The defining feature of ER is the activation of a goal to **influence the emotion trajectory**.	
	Hervás (2011)	To modulate the emotions we need to be openned to, attend, label, accept, analyze the origin and message of the emotions. We need to determine if the message is valid or a false alarm.	Strategies of ER need some indicator that allows us to classify strategies in functional or non-funciontal.
The **content** of emotional regulation (why people regulate their emotions)	Tamir (2016)	ER is a motivated process; **the establishment and pursuit of goals** shape emotional regulation. **Taxonomy of goals of ER** [hedonic (prohedonic, counterhedonic) or instrumental (performance to do, epistemic to know, social to relate, eudaimonic to be)]	

The bold text aims to emphasize and highlight the central concepts proposed by each author from their perspective on emotional regulation.

Gross’s (1999) model follows the sequence situation-attention-appraisal-response:

Emotion begins with an evaluation of emotion cues. When attended to and evaluated in certain ways, emotion cues trigger a coordinated set of response tendencies that facilitate adaptive responding. These responding tendencies involve experiential, behavioral, and physiological systems. Response tendencies from each system may be modulated, and it is this modulation that gives final shape to the manifest emotion (p. 528).

Emotion begins with an appraisal, followed by a set of responses that can be modulated. This latter concept refers to emotional regulation, a process that can be defined in various ways. According to Thompson (1991), the concept of emotional regulation refers to “the extrinsic processes responsible for monitoring, evaluating, and modifying emotional reactions, especially their intensive and temporal features, to accomplish one’s goals” (pp. 27–28). According to Gross (1999), emotional regulation refers to “those processes by which people exert an influence on the emotions we have, on when we have them, and on how we experience and express them” (p. 275). Both authors elaborate a joint definition of emotional regulation, referring to the individual’s efforts, conscious or unconscious, to influence at some point in the process of emotion generation (Gross and Thompson, 2007). Subsequently, Gross et al. (2011) emphasize that the defining feature of emotion regulation is the activation of a goal to influence the emotion trajectory. Therefore, a goal is necessary to carry out such emotional regulation, an issue that will be further explored later and more directly in the research by Tamir (2016) and which is especially relevant when considering the relationship between emotional regulation and flourishing, as we will see below.

To carry out such emotional regulation, there is a wide variety of strategies, which can focus on different aspects of the emotional process. Despite the variety of instruments and categorizations of these strategies, some authors conclude that in practice they comprise the same type of emotional regulation strategies (González et al., 2006). These strategies are focused on modifying what we feel (Pascual Jimeno and Conejero López, 2019).

Gross recognizes reappraisal as the deliberate strategy par excellence to regulate emotion, since it allows modifying the valuation given to the stimulus that generates the emotion (Etkin et al., 2015) and allows changing the way of thinking about a situation to reduce the emotional impact it produced (Gross, 2002). Nonetheless, there are other strategies available. The core feature for this author is the adaptiveness or functionality of these strategies. This notion of adaptive or maladaptive is based on evolutionary psychology and does not fully fit into our proposal as we will explain afterwards.

Hervás and Vázquez (2006) refer to Gross and Thompson definitions of emotion regulation and focus on some limitations of the Gross model: the lack of emphasis on emotional acceptance which is crucial to develop acceptance toward emotions and serves as a mechanism of emotional regulation; the risk of using emotional regulation as a way of evitation, which has been criticized by subsequent research; the difficulty of reevaluating or changing significance in some situations. They conclude enlightening that this model does not distinguish with detail which options of emotional regulation are adaptive and functional or dysfunctional and disadaptative.

For this reason, Hervás (2011) proposes a model of emotional processing consisting of six stages: emotional openness, emotional attention, emotional labeling, acceptance, emotional analysis and emotional regulation. We will focus on the last stages (emotional analysis and emotional regulation) because of the link between these stages and Arnold’s proposal. Hervás proposes four element keys for analyzing emotion: origin, message, validity and learning. He proposes to recognize the origin of the emotion and to understand the message, the meaning of such emotion, as well as he points out that it prepares us to respond. Subsequently, he indicates that the signal offered by emotions can be “right or wrong” and that this analysis corresponds to the person: to contrast the situation as objectively as possible and decide whether the emotion is a valid message or a false alarm. For this analysis, he proposes cognitive strategies such as the evidence search technique, the double parameter technique or the pie technique. If the emotions are valid, it will be necessary to draw the relevant conclusions, learn for the future and develop an action plan. After this learning, Hervás proposes the function of emotional modulation, which is the capacity to modulate the emotional response through emotional, cognitive or behavioral strategies.

This analysis elaborated by Hervás offers valuable insight into the concept of emotional regulation: there is no indicator that allows us to classify when and why some strategies are adaptive and functional and when they are not. To carry out this emotional processing we need additional information that allows us to know when our action is functional and when it is not. According to what we propose in this article, this information would come from the concept of flourishing.

Subsequently, Tamir (2016) highlights that research in emotional regulation has primarily focused on the *process* of emotional regulation and has only recently raised questions about the *content*, such as why people regulate their emotions and what they want to feel. This author confirms the relationship between the motives and goals of emotional regulation and offers a taxonomy of motives in the field of emotional regulation: hedonic (prohedonic, counterhedonic) or instrumental (performance *to do*, epistemic *to know*, social *to relate*, eudaimonic *to be*).

In the same vein, Tamir et al. (2020) state that emotional regulation is a motivated process and that the establishment and pursuit of goals shape emotional regulation; it is necessary, among other aspects, to promote adaptive emotion regulation. They also assert that the relationship between emotional regulation and well-being is complex, as emotions serve as goals to achieve but also as signals of progress toward the set goal. Therefore, they suggest that future research should focus on revealing how goal setting and goal striving jointly contribute to adaptive functioning. This point is of particular relevance for understanding the intimate connection between emotional regulation and motivation, which will also be addressed by Arnold, and to explain how flourishing is reflected in emotional regulation. Next, we will delve into what flourishing is in current psychology and present the research that links emotional regulation and flourishing.

## 3 Flourishment

The topic of flourishing has been of interest in psychology over the past few decades and the number of research studies in this area has been increasing over time (David et al., 2013; Vittersø, 2016c; Cherkowski and Walker, 2018; Diener et al., 2018; Galvin, 2018). This surge can be attributed, among other factors, to the widespread concern in some prosperous societies about being happy, an issue addressed by health sciences and social sciences, and the emergence and promotion of Positive Psychology, which aims to promote human flourishing and thereby happiness (Lopez and Snyder, 2009; Brown et al., 2018). The study of this extensive psychological research on flourishing leads to three related findings: there are multiple conceptions of flourishing, it is designated by different terms, and there are different instruments and constructs for measuring flourishing. Measuring human flourishing is essential to drive research and guide psychological intervention related to both mental health and personal development. Some researchers have recently emphasized the need for a coherent theoretical foundation and a comprehensive approach to flourishing that serves to improve and complete current measurement instruments (Fowers et al., 2023). Given this state of the art, we pose these questions: What conception of flourishing does current psychology research tend toward? What aspects of these theories could be illuminated by Arnold’s theory on emotional regulation and the self-ideal?

### 3.1 Other ways to define flourishing

The first step is to provide an explanation of the different names for flourishing. Although it is frequent in the literature to resort to different nouns to designate the same reality, it also happens that the introduction of a different name than usual marks the meaning of the reality. Both situations occur in the science of flourishing. The literature expresses the reality of flourishing with different names, which from the oldest to the most recent nomination are: eudaimonia, good life, happiness, well-being or wellbeingor flourishing. Eudaimonia is good life, fulfilling life, self fulfillment, and it has its origin in Aristotelian philosophy (Dabdoub et al., 2020). Eudaimonia is translated as happiness, “a complete life that goes well for the person leading it” (Vittersø, 2016a, p.1) although over time it has come to mean feeling good with a full or good life, to later reduce the meaning of being happy to only feeling good-positive emotions, life satisfaction and pleasure-. Wellbeing is the term that refers to that sense of happiness that reflects a state of mind, a being well because of feeling good (Diener et al., 2018). With the word flourishing, the meaning of eudaimonia is recovered, which includes feeling good, being and doing well. In these conceptualizations, emotions and actions to achieve happiness or have a happy life play an important role (Intelisano et al., 2020).

### 3.2 Theories on flourishing

The second step is to delve into psychological theories on flourishing, which in most cases are made and remade, based on the results of empirical research. Exploring the content of instruments measuring flourishing, we discover the ideas researchers hold about what human flourishing entails. Fowers et al. (2023) highlight eight measures of flourishing. The authors analyze the consistency of the psychological theories upon which the constructs are built and conclude that there is a lack of a coherent theoretical foundation on flourishing. The instruments they refer to are:

Psychological Well-Being Scale (Ryff, 1989), Mental Health Continuum (Keyes, 2002), Flourishing Scale (Diener et al., 2010), the Questionnaire for EudaimonicWell-Being (Waterman et al., 2010), Positive Emotion, Engagement, Positive Relationships, Meaning and Purpose, and Accomplishment (Seligman, 2011), Flourishing (Huppert and So, 2013), Comprehensive Index of Thriving (Su et al., 2014), and the Flourishing Index (Vander Weele, 2017) (Fowers et al., 2023, p.123).

The constructs that serve as the basis for establishing the structure and domains of measurement instruments ultimately form the content of diverse conceptualizations of flourishing in psychology. If instruments measuring happiness capture the degree of satisfaction, life satisfaction or well-being based on feeling good (pleasure, emotions and positive affects), they are conceiving the construct of psychological wellbeing (PWB) as a subjective well being (SWB) or hedonic well being (HWB). But if in the instruments introduce other domains related to having other goods that make up a complete life, then PWB is identified with eudaimonic well being (EWB), an objective well being: that which makes one well and makes all people well. There are two main conceptualization paths of EWB: one focuses on objective elements of psychological functioning, while the other adds to it, including the pursuit and achievement of the true self, the self-realization, the pursuit of excellence. In all cases, flourishing has been operationally defined by the degree and frequency of well-being indicators, how individuals feel and their level of satisfaction, both in themselves and in relation to the goods, experiences, activities and qualities reflected in their lives. Fowers et al. (2023) criticize the difficulties presented by measurement instruments precisely because they follow this assessment path, in addition to presenting other fragile aspects such as the underlying notion of flourishing, as we have highlighted in previous lines, or whether they are valid instruments for populations from different cultures.

The tendency to conceptualize flourishing as EWB in psychological research, with an integrative view of the elements contributing to well-being-both in terms of feeling and condition (Ryff et al., 2021) shows an approach to the Aristotelian notion of Eudaimonia (Waterman et al., 2010). The concept of flourishing, which has been developed especially in the last 20 years in the context of Positive Psychology, is often denied as the optimal state of well-being (Richard-Sephton et al., 2024). It comprises three dimensions: SWB or HWB, PWB or EWB and social well-being. SWB results from the predominance of positive emotional experiences (experiences and judgments of satisfaction) over negative ones. PWB stems from the ability to lead a meaningful and purposeful life, with autonomy. Social well-being depends on relationships with other people and with the community. The concept of well-being and, consequently, of flourishing, is distinct from that of mental health, which denotes the absence of negative features in functioning, emotions and behavior, which prevent the individual from realizing their potential and connecting with their environment (work, family and community) (Richard-Sephton et al., 2024). This psychological concept of well-being as optimal functional psychological development aligns with part of the content of eudaimonia or flourishing in its Aristotelian sense and is also embraced in educational theory: “the actualization of human potential so that each person leads a good life” (Martínez et al., 2023, p. 23). In the words of Kristjánsson (2020), p. 23:

Progressive development of subjectively determined and objectively valuable meaningful life, which satisfactorily mobilizes the individual's natural capacities in areas linked to specific and existential tasks of their species, in which humans as rational, social, moral, and emotional beings can achieve excellence.

Vittersø (2016a), p. 8 states that the most representative theories of the literature on psychological eudaimonics are: “(..) Waterman’s Eudaimonic identity theory (Waterman, 1984; 1992), Ryff’s version of Psychological well-being (Ryff, 1989) and Deci and Ryan’s Self determination theory (SDT; Ryan and Deci, 2001)”. These theories focus on an aspect of human flourishing while considering Aristotle’s theory of eudaimonia: the fulfillment of life as the ultimate purpose of being human, an optimally functioning life. In addition to these theories, Fowers et al. (2024) have proposed a systematic theory of flourishing from a neo-Aristotelian approach that inspires and provides a foundation for psychological research on the topic. They suggest taking into account eudaimonia with a broader perspective, a vision of what contributes to the process of living well and the outcome of having a good life. To do this, they include more elements than those promoting the development of all necessary capacities to achieve optimal psychological functioning and assert that harmony among them is important.

We believe that Arnold does indeed have in mind this comprehensive framework of what flourishing is because she is knowledgeable about and grounded in Thomistic philosophy, which has an Aristotelian basis:

We must organize our personality according to a valid self-ideal if we are to escape the consequences of emotional indulgence. Contradicting the demands of our nature by giving free rein to our emotions instead of allowing them to support our human purposes inflicts us with a penetrating discontent and an unspeakable anguish. Aspiring to a self-ideal that does not perfect human nature is not worth the effort. To suppose that human nature can withstand the permanent frustration of its deepest desires is to seek fundamental anguish (Arnold, 1970, p. 313-314).

It is precisely this vision that underlies her theoretical proposals on emotional regulation and the self-ideal, which we believe can inspire intervention models to promote people’s flourishing, as seen in the final section of this article. The construct of flourishing has its roots in a classical concept, impossible to operationalize in all its breadth and depth, which is the concept of happiness. The concept of happiness is based on conceiving fulfillment in the unfolding of a person’s potentialities and is recognized in various ways in different cultures, philosophies, and religions.

The most frequently referenced concept is the Aristotelian notion of *eudaimonia* (εὐδαιμονία). Aristotelian eudaimonia is a state of full deployment of human operational capacities, whose core is the knowledge of truth but also requires the presence of other goods, its indispensable conditions, such as possessions, health, ethical virtue, and, above all, friends, and would result in a subjective state of joy. From this point of view, eudaimonia is not only PWB, but also the other forms of well-being (subjective and social) that would be either its conditions, or its consequences. Thus, the Aristotelian concept of eudaimonia coincides with the construct of flourishing. This coincidence is significant because when Arnold speaks of happiness, she has in mind the Thomistic concept of *beatitudo*, which is Aquinas’ designation for what Aristotle calls *eudaimonia*, another author whose contribution to the concept of flourishing has been highlighted (Titus, 2016).

### 3.3 Contents of flourishing

The third step is to examine the elements that constitute flourishing or a fulfillment life according to the scales highlighted by Fowers et al. (2023), from which we discard two closely related to mental health. The selected scales draw on many cases from the three major theories of psychological eudaimonics (Vittersø, 2016a). These instruments coincide in some domains, as seen in Table 2, and those that are not common are related. Although all of them aim to assess psychological functioning, they include domains that are closer to a eudaimonic conception of flourishing, with the content attributed to eudaimonia by Aristotle.

**Table 2:** Measures of flourishing.

Psychological well-being (Ryff, 1989)	Social-psychological prosperity or psychological flourishing (Diener et al., 2010)	Comprehensive inventory of thriving (Su et al., 2014)	PERMA profile scale (Seligman, 2011)	The flourishing index (Vander Weele, 2017)	Questionnaire for Eudaimonic well-being (Waterman et al., 2010)
Self-Acceptance Positive relations with others, Autonomy, Environmental mastery **Purpose** in life Personal growth	**Relationships supportive** Engaged Contribute to others **Purpose** and **meaning** Competence Good person Optimistic Respected Positive feelings Negative feelings Balance feelings	**Relationships** Engagement Mastery, Autonomy, **Meaning**, Optimism, Subjective WB	Positive Emotion, Engagement **Positive relationships** **Meaning/** **purpose** Accomplishment	Happiness and life satisfaction Health, both mental and physical **Meaning and purpose** Character and virtue **Close social relationships**	Self-discovery Perceived development of one’s best potentials, **Purpose/ Meaning in life** Investment of significant effort in pursuit of excellence Intense involvement in activities Enjoyment of activities as personally expressive
Psychological functioning and health mental (PWB)	Social–Psychological functioning and WB (PWB + SocialWB+SWB)	Psychological functioning and health mental (PWB + SWB)	Flourish and WB (SWB + PWB)	Flourishing (SWB + PWB + EWB)	Eudamonic WB (SWB + PWB)

The data were obtained from different sources (Ryff, 1989; Diener et al., 2010; Waterman et al., 2010; Su et al., 2014; Vander Weele, 2017; Carvalho et al., 2021; Fowers et al., 2023).

The inclusion of domains characteristic of SWB is present in all instruments except Ryff’s (1989): SWB experiences of eudaimonia/feelings of personal expressiveness; positive feelings, negative feelings, balanced feelings, positive emotion, happiness, and life satisfaction, enjoyment of activities as personally expressive.

The construct present in all instruments is purpose (in life) and meaning (in life), highlighted in Table 2 in bold. Meaning is the significance and coherence of one’s life, or sense of one’s life, “having a deep, coherent, organizing conceptual framework for one’s life, one that helps define who we are as individuals and what is most important to each of us (Danvers et al., 2016) or “the fulfillment of the intrinsic values of our human nature” (Vittersø, 2016a, p. 5). Meaning gives sense to purpose, which is the end that people seek when directing their lives, which is reflected in the goals they pursue to achieve that end. A person who reasonably defines his or her own framework of meaning, chooses values and/or goals. Making meaning of one’s life is a basic human need, satisfying it is an indicator of flourishing, as is also directing one’s behavior according to purpose, values and goals. Flourishing is supported by a meaning that exists along a continuum in life. Researchers explaining the relationship of this construct with flourishing or PWB mention the role of feelings, emotions, and affects, not only to assess if people feel good but also as essential elements for having meaning and purpose.

Relationships with others: positive relations with others, supportive relations (supporting oneself and supporting others), contribute to others, relationships (in two instruments), close social relationships, are the constructs mentioned in all the instruments (Table 2, highlighted in bold) except in Waterman et al.’s (2010). Relatability with others is a basic psychological need. Affectivity also plays a relevant role in achieving relationships with others, while also reflecting whether they contribute to WB. Building positive and supportive relationships can be a goal that is part of life purpose and meaning.

The instruments contain various constructs that are related to the capabilities and qualities of people, which demonstrate that the person has achieved or is achieving human flourishing understood as an optimal psychological functioning. We refer to: self-acceptance (self and past life); autonomy (in two instruments); environmental mastery (adapting to an environment), mastery (dominance), accomplishment; competence (ability to act), optimistic (in two instruments), engaged and engagement (in two instruments), respected, health, both mental and physical.

Other domains such as character and virtue, good person, personal grow, the pursuit of excellence and self-realization, self-discovery, perceived development of one’s best potentials, Intense involvement in activities, indicate the process of flourishing and the outcome achieved in that process: being virtuous, being a good person, being oneself.

The domains demonstrate a SWB (feelings and satisfaction life), a PWB (psychological functioning) and a Social WB (relationships with others), all of which added together is what is contained in an EWB. However, as pointed out more clearly by Waterman et al. (2010) and Vander Weele (2017) when arguing about the theoretical foundations of the instruments they have developed to measure flourishing and the most prominent psychological theories of flourishing, Waterman’s Eudaimonic identity theory (Waterman, 1992), Ryff’s version of Psychological well-being (Ryff, 1989) and Deci and Ryan’s Self determination theory (Ryan and Deci, 2001), functioning well, in the sense of efficacy, is not in itself the end that is identified with having a complete, fulfilling, or good life. This is explained by Waterman et al. (2010, p. 6):

The theory links eudaimonist philosophy with the study of psychological functioning, and it emerged from consideration of two questions. (1) In the task of identity formation, do some potential identity elements represent ‘better’ resolutions to an identity crisis than others? (2) If so, how are the ‘better’ choices to be recognized? Eudaimonic identity theory draws upon eudaimonist philosophical constructs, including the daimon or ‘true self’, self-realization, the pursuit of excellence, and eudaimonia (..).

The process of identity formation, process of self-determination includes the consideration of values, self or personality (Bauer, 2016; Schlegel et al., 2016) and life (Dahla et al., 2020) concretized as goals to be achieved, thus intrinsically motivating action. Feelings, emotions and experiences of satisfaction are indicators of success in the choice and execution of values and goals in life, but they also play an active role, aligned or not with motivation (Waterman et al., 2010; Danvers et al., 2016; Vallerand, 2016; Vittersø, 2016b; Chaves, 2021).

## 4 Relationship between emotion, emotional regulation and flourishing

In the previous sections, we have discussed flourishing and emotional regulation. The complexity of the construct of flourishing has been highlighted. In an initial stage of research, flourishing is understood as the optimal state of well-being and has been operationally defined by the degree and frequency of well-being indicators (Richard-Sephton et al., 2024). In a later stage, the meaning of flourishing is expanded, drawing inspiration from Aristotle’s conception of eudaimonia. Emotional regulation, in turn, designates a psychological and behavioral process of identification, monitoring, evaluation, and adjustment of one’s emotional response to events and circumstances internal and external to the individual. If SWB or EWB is considered an integral part of the flourishing construct, understanding SWB as the predominance of positive emotional experiences (experiences and judgments of satisfaction) over negative ones (Richard-Sephton et al., 2024), it becomes evident not only that there is a connection between emotion and flourishing, but also that there seems to follow from this a connection between emotional regulation and flourishing. Indeed, emotional regulation appears to be a process that enables directing the emotional experience toward that experience of high well-being that would be flourishing.

If adequate emotional regulation promotes greater well-being and a more fulfilling life, research should be able to establish a positive correlation between both constructs. Although the results may sometimes be more ambiguous than expected, in general, such a positive correlation is observed. Research has focused on determining the most effective strategies to promote flourishing (Barber et al., 2010) and on the correlation between the use of the emotional regulation strategies most known by the scientific literature and flourishing (Richard-Sephton et al., 2024). As we have pointed out earlier, these researchers establish that emotional regulation strategies can be adaptive and functional or maladaptive and dysfunctional. Adaptive and functional strategies are useful strategies, as they help the person successfully achieve the desired emotional state. Adaptive strategies promote positive emotions; functional strategies aim to decrease negative emotions. On the other hand, maladaptive and dysfunctional strategies would be useless strategies, as they are ineffective in achieving the desired emotional state or because they are associated with emotional or psychological difficulties. Maladaptive strategies decrease positive emotions. Dysfunctional strategies increase negative emotions.

Rumination, avoidance, and suppression are three common types of emotional regulation strategies that are associated with poor mental health outcomes. We attempt to link emotional regulation strategies with flourishing. *Flourishing* is associated with increased use of cognitive reappraisal, which is the fundamental adaptive strategy, and with decreased use of experiential suppression or avoidance (Barber et al., 2010; Richard-Sephton et al., 2024). Overall, it is observed that *flourishers* report lower use of maladaptive strategies than significant use of adaptive strategies, compared to non-pathological individuals who are not flourishing, highlighting the positive use of reappraisal. Similarly, rather than showing a significant difference in the use of functional strategies to minimize negative emotions, there is a lower use of dysfunctional strategies that exacerbate negative emotion (Richard-Sephton et al., 2024). The results, therefore, demonstrate a correlation between emotional regulation and well-being or flourishing, but more clearly due to the lack of resorting to maladaptive and dysfunctional strategies than due to a significant difference in the use of adaptive and functional strategies, fundamentally highlighting the use of reappraisal.

These results require a theoretical elaboration that allows accounting for the different aspects and adequately addressing the theoretical questions they raise. We may ask what makes an emotional experience positive or negative, and whether it is appropriate to call adaptive and maladaptive, functional and dysfunctional strategies. If we speak of a connection between emotional regulation and flourishing, perhaps the word adaptation, so closely related to the purpose of survival, may not be the appropriate word. Even more so, this inadequacy can be considered from the perspective of flourishing understood as eudaimonia, which does not only consist of surviving but of living well. Therefore, the concept of eudaimonia needs to teleologically influence the concepts by which an emotion is valued.

On the other hand, a theoretical answer is needed to the question of how flourishing relates to emotional regulation that, beyond correlation, points to causes. The presence of a cognitive cause is evident, since mechanisms such as reappraisal are cognitive strategies of emotional regulation. This is where Magda Arnold’s concept of self-ideal and its role in emotion regulation can play a key role. On the one hand, if the emotion is caused by an intuitive evaluation, it is about seeing how that intuitive assessment relates to intellectual and reflexive cognition (Echavarría, 2020), a topic on which Arnold explicitly focuses. In turn, the self-ideal allows for a pivot between these levels of cognition and promotes a synthetic grasp of the state of fulfillment designated in current research and theory with the name of flourishing.

## 5 An integrated conceptualization of emotional regulation and flourishment

**Figure 1:**
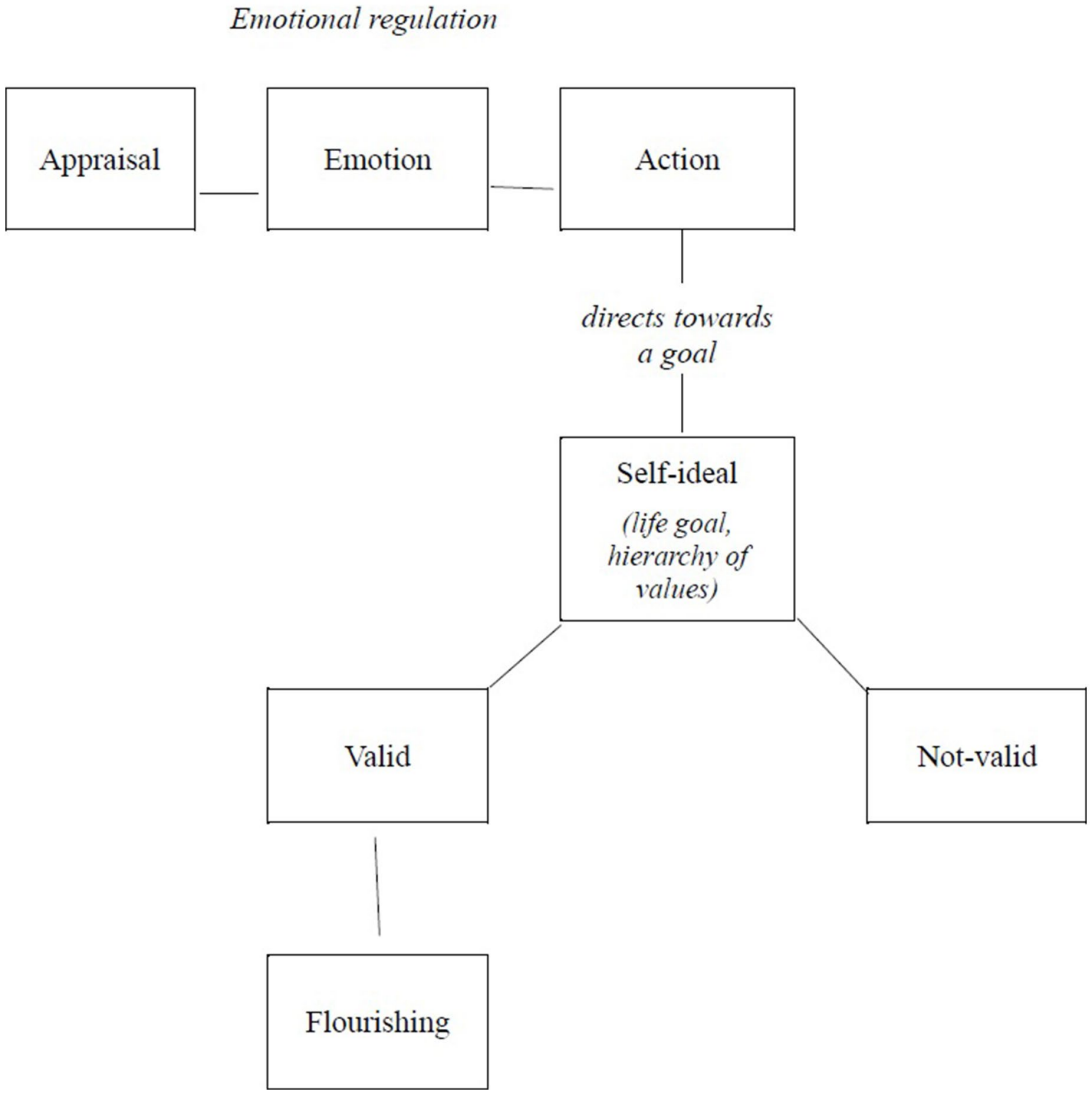
Relationship between emotional regulation, self-ideal, and flourishing. Table created by the authors based on Arnold’s theory (Arnold and Gasson, 1954; Arnold, 1970, 1984).

After defining the concepts of emotional regulation and flourishing and reviewing the state of the art in the relationship between both concepts, we proceed to present a theorization that integrates both concepts, taking as a starting point the notions outlined in Arnold’s theory. It is relevant to highlight that the terms used by this psychologist differ slightly from those used today; nevertheless, we believe that what matters are not the terms used but the concepts to which they refer. Understanding these concepts is precisely what will allow us to offer an integrative conceptualization that combines subsequent research on emotional regulation and its relationship with flourishing. Figure 1 includes a diagram that synthesizes this integrated conceptualization.

To focus our analysis, we will address the following issues: (a) the link between appraisal, emotion and self-ideal; (b) the concept of self-ideal; (c) the importance of the self-ideal in emotional regulation; (d) emotional regulation strategies; (e) the relationship between emotion and motivation.

### 5.1 The link between appraisal, emotion and self-ideal

Arnold is known for being a pioneer in the theories of appraisal in emotion psychology. Her concept of appraisal inspired Lazarus, a renowned psychologist in this field. In an article published in 1968, Lazarus acknowledged Arnold’s influence: “the [present] view of emotions which emphasizes cognitive processes as antecedents and the arousal of coping impulses to deal with appraised danger is an elaboration of that presented by Arnold” (Lazarus, 1968, p. 190 cited in Reisenzein, 2006, p. 940). Likewise, Lazarus (2001) pointed out the differences between Arnold’s concept of appraisal and his own and described how this concept, first used by Grinker and Spiegel in 1945 but without a specific meaning, is conceived by Arnold fundamentally as an instantaneous, intuitive, and automatic process. Like Arnold, Lazarus distinguished between primary and secondary appraisal, non-conscious and conscious, but while Arnold focuses on the former, with an intuitive and immediate character, Lazarus focuses on the latter, on the reflective process and its dependence on complex meanings.

Arnold proposes the following sequence: perception - appraisal - emotion. Emotion is defined by Arnold (1969a,b) as a felt tendency toward an intuitively valued object as beneficial or away from an object appraised as harmful. This way of understanding emotion is very similar to that later developed by Gross (1999, 2015), a reference psychologist in the field of emotional regulation. Therefore, emotion stems from an evaluation of reality, from an appraisal. The evaluation preceding the emotion provides information about what is important to the individual, about what they value, according to the meaning that a situation or object has for them. Arnold (1969b) distinguishes between intuitive and reflective evaluation, explaining the difference through the example of a man suffering from obsessive-compulsive disorder due to the fear of contamination, which leads him to wash his hands incessantly every time he touches something. This fear of contamination and germs causes him to wash his hands even though his conceptual and reflective knowledge tells him that his fear is exaggerated and that his skin can tear with frequent washing. In this case, intuitive evaluation produces fear and reflective evaluation makes him aware that he is powerless against that fear. This deliberate judgment is conscious, and intuitive evaluation, on the contrary, is not experienced consciously, although it can be inferred through reflection. Both appraisals are value judgments that do not necessarily lead to action, but they can do so. In this sense, Arnold asserts that each person establishes a hierarchy of values for themselves that guides their actions, which she calls the self-ideal. This guidance can lead to their development and perfection as a human being or move away from them.

According to Arnold, when we appraise something we also value the extent to which it contributes to or detracts from the ideal we aspire to, which she calls the self-ideal: “Whenever anything seems attractive or pleasant, it is also appraised as it contributes to or detracts from the ideal toward which he aims” (Arnold, 1970, p. 302). In this way, Arnold introduces a novel aspect into her concept of emotion, not considered by other authors, which is its relationship with the self-ideal. She defines it as the ideal that guides the person’s action and organizes his or her personality. Therefore, personality is shaped around the attainment of this ideal, which constitutes the direction or goal around which their actions and abilities are organized. Arnold describes emotions as “guardians of the self-ideal” since they can help the person on their path toward the self-ideal: negative emotions lead the person to change their way of acting, and positive emotions facilitate following the valid self-ideal.

According to Cornelius (2006), in Arnold’s view, emotion is a reflection of the significance that the situation or object holds for the person, and not knowing what a person values, their self-ideal, can hinder understanding why they value objects and situations the way they usually do and, therefore, why they feel what they feel in a particular situation.

In this sense, Arnold asserts that the self-ideal a person has developed assigns value to what they encounter. At this point, it is interesting to note the difficulty we encounter in distinguishing in Arnold’s proposal whether that value refers to intuitive or reflective appraisal, especially in cases where they differ. According to the analysis of her complete theory, we could conclude that there is a relationship between reflective value judgment and the person’s self-ideal, but we cannot forget the emotional residue (or affective memory) left by various emotional experiences, which affects intuitive appraisal, leading to reactions that do not align with rational judgment and can hinder the path toward the self-ideal.

### 5.2 The concept of self-ideal

Arnold does not offer a single definition of the self-ideal, but throughout her writings, she refers to this notion in various ways: as a “life goal,” “what we (..) are striving for,” “what, in striving, we finally achieve” (1959, p. 34), “the best that is possible for this individual to achieve,” “the perfection both of his individuality and his humanity.” Therefore, the self-ideal is a life goal, an ideal that consists of achieving perfection. Furthermore, according to Arnold, the self-ideal guides the organization of personality: “These rational tendencies to action organize human personality under the guidance of the self-ideal” (Arnold, 1970, p. 295); “We must organize our personality according to a valid self-ideal” (p. 313), she also includes it in her definition of personality: “the human being can and does organize his powers, actions, and habits in the active pursuit of his self-ideal” (1959, p. 33). Therefore, the self-ideal is not only a goal but also a guide and organizing principle.

Arnold assumes that the self-ideal is formed and that not any self-ideal will suffice; Arnold distinguishes the valid self-ideal because it reflects such perfection: “a valid self-ideal is the perfection of a man’s humanity,” “a self-ideal that is objectively valid and that represents ideal humanity as it can be achieved by this particular individual” (Arnold, 1970, p. 306). Therefore, the self-ideal is not purely subjective but has an objective dimension.

The human ideal, therefore, relates to that of perfection: “self-ideal is the perfection of a man’s humanity” (Arnold, 1969b); “human perfection must be found in a self-ideal that is formed according to the best that a man knows and understands and in actions that will actualize this ideal” (Arnold, 1970, p. 302), and therefore, an indication of maturity is that a self-ideal has been elaborated: “A man’s self-ideal is the index of his maturity” (1959, p. 34); “maturity means forming a valid self-ideal and living it.”

On the other hand, Arnold relates the self-ideal to happiness, which she understands as “happiness is the state of a person who has chosen a self-ideal appropriate to his human personality and has steadfastly followed it” (Arnold, 1969b); “The desire for happiness, even more than other positive human emotions, urges us to maintain a straight path toward our self-ideal” (Arnold, 1970, p. 344). Therefore, happiness allows one to approach perfection or the self-ideal, just as happiness is a sign that one has chosen the appropriate self-ideal.

Additionally, the self-ideal in Arnold’s theory is closely related to values: “Thus, a man’s self-ideal is an index of his maturity, for it reveals his scale of values” (1970, p. 297), “as soon as the child’s self-ideal begins to form, it becomes a touchstone for everything else to which he attaches value” (p. 300). That is, it manifests the person’s hierarchy of values.

Moreover, the self-ideal triggers a hierarchy of motivations: “Motives are ordered into a hierarchy according to what is, by and large, the most important goal. (..) the master goal becomes our master motive, the self-ideal that shapes us as we strive toward it” (Arnold, 1970), “Therefore, a man’s motivational system is established and organized around his self-ideal” (Arnold, 1970, p. 302), “a hierarchy of motives gradually develops as the child begins to understand what is more and what less important” (Arnold, 1959, p. 34).

In summary, Arnold explains the relationship between values, motivation, and the self-ideal with these words: “Such rational choice of action eventually establishes a hierarchy of values: What is valued most is what we want so intensely that we are willing to forego every other pleasure or satisfaction rather than lose it. This most wanted thing is our life goal” (1959, p. 34). Thus, she considers that what is valuable becomes a motive when it moves us to act, and this shapes the hierarchy of values reflected in the self-ideal.

### 5.3 The importance of the self-ideal in emotional regulation

Arnold argues that although emotion is necessary to prompt action, it does not determine human behavior. In other words, humans always have a range of options in decision-making, the capacity for self-determination, and the ability to exert executive control over psychological functions (Arnold, 1984). Therefore, emotion does not necessarily propel a person toward self-improvement: it can either lead them toward the self-ideal or not, so it cannot be relied upon as a guide:

Human beings are motivated by an appraisal that is both a sensory judgment and an intellectual or reflective judgment. The final decision for action is a choice that either implements the original emotion or opposes it. In man, the choice of goal-directed action is essentially a rational wanting, an inclination toward what is reflexively appraised as good (pleasant, useful, or of value). These rational tendencies to action organize human personality under the guidance of the self-ideal (Arnold, 1970, p. 295).

This goal is achieved when emotional responses are guided by higher ideals, harmonizing emotional reaction with our intellectual apprehension of the world (Cornelius, 2006). Therefore, the appropriate self-ideal sets the direction for action, which in more contemporary terms, we could call flourishing or eudaimonic well-being.

In this regard, we highlight the contribution of Valenzuela (2024), who relates positive and negative emotions, as conceived by Arnold, to the concept of flourishing:

Various ‘positive’ emotions (interest, union with others, love, joy, (…) can make it easy to seek the ‘self-ideal’, that is, the flourishing or maturity of one’s personality. (…) negative emotions (fear, shyness, embarrassment) which hinder reasonable action-and keeps us from flourishing). (p. 304-5).

What role do emotions play on the way to this flourishing? According to Arnold (1970) all emotions can help a person to move toward his or her self-ideal: negative emotions arise when something has been done that is valued as wrong and urge the person to repair his or her action and change his or her way of living and positive emotions sustain this progress toward the self-ideal, attracting toward the beneficial or moving away from the negative and, on the other hand, they urge to strive to achieve it, overcoming the possible difficulties in this process, or moving to escape from dangers. Therefore, Arnold uses the term negative and positive when talking about emotions, referring to the extent to which these emotions bring us closer to or prevent us from moving away from the self-ideal. However, sometimes emotions can drive us to changes that are not constructive or it can happen that the self-ideal is not the right one, so that emotions move the person to act, but are not necessarily directed toward the flourishing of the person.

The appraisal preceding the emotion provides information about what is considered good or bad at a given moment. However, as mentioned earlier, this appraisal may be conditioned by our affective memory and may not accurately reflect the present reality or object (Arnold, 1969a). Therefore, following Arnold’s reasoning, emotion cannot be relied upon as a guide to achieve maximum integration and perfection.

Moreover, when this ideal is mistaken or adapted more to one’s own utility, conflict, dissatisfaction, and discomfort will arise. Arnold distinguishes between acting against an objectively valid self-ideal, which generates remorse and leads to wanting to regain the correct direction, and possessing a distorted ideal that does not fulfill all of a person’s potentialities. In the latter case, there would be no remorse for acting inappropriately. However, in this situation, an unconscious conflict would arise between the path chosen by the individual and the inherent tendency toward self-perfection in their nature (Arnold, 1959, p. 35). It may also happen that an emotion goes against the person’s reflective tendencies and deliberate purposes, thereby hindering action in that direction, especially if an emotional attitude or habit has developed. Excessive or chronic emotion can alter psychological functioning (Arnold, 1970).

Therefore, Arnold suggests the need for some control over emotion: “Such control does not mean that emotions should be reduced or restricted, nor that the actions they prompt should be omitted. Rather, emotions should be controlled in such a way that they aid rather than hinder personality organization. (..) Obviously, such control of emotion also implies a worthwhile self-ideal to provide a focus for a man’s struggle” (Arnold, 1970, p. 292–3). In this sense, according to Arnold, the existence of a self-ideal is necessary to guide emotional control. In addition to this control, she also proposes different ways to correct affective memory or, in other words, the reappraisal of past experiences so that it aligns with the reality in front of us: corrective experiences and reflexive reappraisal.

Both strategies are proposed by Arnold for regulating emotions. However, she never mentions the terms emotional regulation or management, concepts used today, but rather focuses on understanding that emotion can help achieve one’s own self-ideal but can also hinder it. In this sense, she does not advocate for suppressing or repressing emotions, which is colloquially understood as emotional control. Her proposal actually resembles what Gross and Thompson (2007) refer to as emotional regulation, as the process by which the intensity, duration, magnitude, and responses related to emotion are modulated. The difference lies in Arnold’s reference to the self-ideal. Therefore, Arnold’s approach goes beyond the notion of adaptation employed by today’s theorists, expanding the goals of emotional regulation.

### 5.4 Emotional regulation strategies

Throughout her work, Arnold explains different ways of regulating emotions. Firstly, she points out the need to recognize the origin of the emotion and to address and confront the situation it presents rather than evade it. In other words, she suggests delving into the information provided by emotions to make constructive use of the acquired knowledge: “For better or worse, emotions influence man’s actions but cannot force them. To derive positive benefits from one’s emotions, a man must recognize their origin and decide to resolve his problem rather than evade it” (Arnold, 1970, p. 323).

Recognizing the origin and meaning of emotions provides necessary information for individuals to make deliberate decisions and persist in the pursuit of the appropriate self-ideal. Understanding the meaning of emotions helps determine what to do. This analysis resembles the emotional analysis phase proposed by Hervás (2011) in his emotional processing model, which suggests analyzing emotions to understand whether the message they convey is valid regarding the reality being reacted to.

Other strategies for regulating emotions employed by Arnold involve increasing the attraction of the goal we want to achieve through imagination or thought: focusing on the positive aspects of something that is feared or disliked, considering ways and means to overcome a bothersome obstacle, exploring alternative courses of action, among other aspects. Thus, according to the goals set by reason and the appropriate self-ideal, individuals can use imagination and thought to impact both reflexive and intuitive appraisal, and ultimately, emotions. Arnold also warns that sometimes, the use of imagination may not be helpful, such as when it increases attraction and therefore the strength of the emotion, as in cases where love or desire for an object becomes excessive. In these circumstances, she suggests finding an occupation that requires the person’s full attention and captivates their entire focus (Arnold, 1969b). Finally, she suggests that at times it may be necessary to deprive oneself of some things that may have great emotional appeal until their influence diminishes.

In any case, a valid self-ideal is necessary to direct a person’s attention toward spiritual values, toward what is more human, and away from objects that exert an excessively strong attraction on the individual without being as necessary or valuable. Therefore, at times, it will be necessary to rework the hierarchy of values and reorganize the goals that drive people.

The difficulty of regulating emotion is heightened when an emotional habit has developed, which requires a strong enough motive to make the decision to act against the emotion. Arnold points out that making the reasonable decision at the beginning of the situation helps reduce the attraction to the opposite course and reduces the pressure that uncontrolled emotion can generate, although emotional inclinations do not disappear during the course of action. The habit of acting for rational reasons, that is, the habit of deliberate choice and rational thought, can serve as substitutes for this emotional habit and provide the necessary strength in the long term to distance oneself from a reality or object that attracts intensely or excessively but is not beneficial or reasonable. In this sense, it is necessary to refer to the Thomistic influence in Arnold’s theory (Echavarría, 2020), which likely understands habit in an Aristotelian sense as a disposition (Hulsey and Hampson, 2014).

Finally, Arnold proposes reflective reappraisal and corrective experiences as tools that allow for the modulation of affective memory. Reflective reappraisal enables a rational reevaluation of the situation experienced. This term is similar to what is known today as cognitive reappraisal. It has been shown that reappraisal is the fundamental strategy employed by flourishers, as mentioned earlier.

On the other hand, corrective experiences are new experiences that, when confronted, allow for the correction of the previous appraisal. Thus, they influence affective memory. This need to influence the emotional impact of memory is also emphasized by Engen and Anderson (2018) as core component processes of cognitive emotion regulation.

Arnold provides a clinical example of a 25-year-old who was still affected by a traumatic experience at 17. It was not a repressed experience, but rather the subject could remember it perfectly well and yet continued to experience excessive anxiety when having to speak with the department head. Arnold points out that he had experienced the same sensation while working for a family friend, upon detecting some irregularities in the business and trying to warn him, but without success. Since then, every time he had to speak with a boss, the severe reaction of fear recurred along with all the physiological symptoms. Arnold describes what happened with this patient and explains how both tools (reflective reappraisal and corrective experience) allow for appropriate emotional regulation:

He had a fixed expectation that every employer would act like his friend and eventually disappoint him. (..) The young man knew he was afraid and recognized upon reflection that this fear (and the accompanying physical symptoms) was unfounded in the current situation; he did not realize that his previous traumatic experience had shaped his current appraisal and intensified his current emotion. (..) Hence, a corrective experience was impossible unless he could realize the connection between his old shock and his current exaggerated fear reaction. In this case, such insight was acquired in a very short time. Above his understanding, there had to be a deliberate decision, supported by his trust in the therapist, to endure the discomfort of these experiences of fear and to act, despite his fear, until he was able to emotionally realize (what he had always known reflectively) that his current employer was not a replica of his former friend. Reflective reappraisal offered him the opportunity to approach the situation with a new attitude until eventually his intuitive assessment was also changed (Arnold, 1969a, pp. 199-200).

### 5.5 The relationship between emotion and motivation

In third place, we will discuss the relationship between emotion and motivation as a basis for understanding the interplay between emotional regulation and flourishing. According to Arnold (1969a), emotion plays a significant role in motivating people to act. A motive, according to the author, is “a want that leads to action” (Arnold, 1971, p. 188). It is, therefore, a desire or a want that drives action and derives from something valued as good or bad, its attractiveness or repulsion to the person here and now (Arnold, 1970). That desire becomes the motive for my actions. The self-ideal developed by the individual organizes their motivational system, which in turn articulates their daily activity (Arnold, 1970). Sometimes, emotion leads to action and becomes the motive for acting. Other times, the person acts for reasons that are not emotional. In both cases, Arnold emphasizes the importance of the person’s motivational hierarchy being in line with a valid self-ideal.

Therefore, we can affirm that not just any goal serves as the objective of regulating our emotions. According to Arnold et al. (1962), there are some motives that are closer to predicting future achievements, performance, or success for the individual, both in school and in life. For this reason, she dedicates her work “Story Sequence Analysis” (1962) to examining people’s motivation:

Would it not be preferable to try for a sample of a man's motives? These, we know, move him to act in distinctive ways. We may then find they reveal creativity, intelligence, aggression, conformity, and any number of other qualities. But, in tapping his motives, we have found the way in which they are combined for action. No longer do we have to be content with disjointed bones in personality analysis. Knowing a man's motives and their hierarchy, we can work with the fleshed skeleton. Thus we will be able, at last, to determine what a person's chances are for achieving excellence. (Arnold, 1962, p. 30).

This motivation is key to achieving what Arnold et al. (1962) calls achievement or excellence. Both concepts are related, although Arnold does not explicitly explain it this way, to the notion of perfection mentioned earlier and, therefore, to the self-ideal. Arnold identifies a type of motivation that correlates with greater achievement. In this sense, we deduce the correspondence between a type of motivation identified by Arnold and an objectively valid self-ideal.

After analyzing the psychological test she developed, Arnold concludes that the type of motivation that leads to better performance includes the following characteristics: it has as its goal immaterial values (ethical, religious, spiritual, and altruistic); it is willing to invest whatever effort is necessary and personal initiative to achieve what is worthwhile; relationships with others are characterized by generosity, cooperation, lasting relationships, and the presence of a common purpose (effort, suffering, or a shared life); adversity is considered to be overcome with one’s own action and a positive attitude, and finally, it includes a specific way of living the relationship with a Supreme Being. In summary, constructive motivation includes taking responsibility for one’s actions, the importance and prevalence of ethical, religious, and altruistic values, and the importance of transcendence (Arnold, 1962). The focus is not only on what a person feels but on the motives that drive them to act.

It seems rather more reasonable to acknowledge that man is responsible for his motives, his intentions, and actions; but he's not responsible for his emotions. He may be so frightened by an early traumatic experience that it would require superhuman fortitude to act courageously in a similar situation. If his motives are positive and constructive, he may nevertheless find ways of controlling his emotion or overcoming it by a corrective experience (see Arnold and Gasson, 1954, p. 92 f.) (Arnold, 1962, p. 221).

Thus, the importance of constructive and positive motives as a means to control or overcome emotion through corrective experiences is highlighted: therefore, the self-ideal is related to emotional regulation. With all this, we can conclude that the relationship between emotion and motivation is key to understanding the relationship between emotional regulation and flourishing. Emotion can be a motive that drives action, but it can also not be. The important thing is that the motivation we have, which helps us regulate emotions, leads us to a valid self-ideal, and therefore, to flourishing.

## 6 Discussion

The study presented in the preceding sections aims to address the primary question posed in the introduction: I How can Arnold’s psychological theory contribute to a better understanding of emotional regulation, flourishing, and the relationship between them?

The path to beginning to answer this question involves considering the relationship between the conception of the self-ideal in Arnold’s psychological theory and current theoretical trends in psychology regarding flourishing. Arnold’s theory represents a fruitful attempt to achieve a comprehensive understanding of human personality. Therefore, it is considered a reference source in the field of psychology, particularly in the realm of emotional regulation. As highlighted earlier, Arnold lays the groundwork for relating personality, emotions, and the self-ideal. The continuity between these aspects is also a characteristic of current theories on flourishing. In our opinion, Arnold forwards, from a more theoretical and general perspective, some inspiring ideas about human development to attain happiness and well-being, ultimately, flourishing.

The reference to the self and its ideal is addressed in research of the last two decades with various terms and meanings. Specifically, the topic of the self-ideal in Arnold’s theory is related to the themes of identity and self; the constructs most frequently used in the literature include: identity (social or moral self), true self (real or perceived), authentic self, true self (Schlegel et al., 2016). The topic of the self is connected to flourishing understood as psychological well-being (PWB) and emotional well-being (EWB) by authors who draw inspiration from the Aristotelian concept of eudaimonia, by researchers who investigate an objective sense of well-being and attempt to develop instruments that measure aspects of well-being beyond just positive emotions and pleasure (Waterman et al., 2010). In broad terms, Arnold’s notion of the self-ideal, a valid self-ideal, emphasizes a type of objective self-ideal. The valid self-ideal consists of humanity fully realized by each individual, their perfection, stemming from their capacity (personality) and circumstances (life history). Arnold refers to a self-ideal that is rationalized as an idea but also manifested in personality and throughout one’s life. The self-ideal is objectively delineated or framed by human nature, “actualized” in each individual by their personal characteristics, actions, and life experiences. The self-ideal is the horizon of good human actions, those that make one fully human and achieve the goods that constitute a good human life.

We believe that some recent research asserts a mode of understanding flourishing that aligns with the conception of the self-ideal. This is evident in the work of Fowers et al. (2024, p. 15), which introduces additional components of flourishing: “We envision a profile approach that will include some currently prominent concepts (e.g., meaning and belonging) and others that have been left out (e.g., harmony with others, harmony with the environment, and collective flourishing).” Up to this point, the objective of flourishing, in the theory that many researchers have worked on, is individual development manifested in good or optimal psychological functioning, necessary for a good life. Arnold situates the self-ideal as something more than individual development; therefore, he conceptualizes it as the organizer of personality. For Arnold:

With Gasson (1954) I would consider personality as the patterned totality of human potentialities, activities, and habits, uniquely organized by the person in the active pursuit of his self-ideal, and revealed in his behavior”. Together with deliberate action tendencies, emotions urge the human being to pursue his ideal. The same combination urges him to aim for particular goals (Arnold, 1969b, p. 196).

For the science of flourishing, the objective of PWB (psychological well-being) and EWB (eudaimonic well-being) is the effective realization of self-actualization by fulfilling basic psychological needs. In this sense, indicators of flourishing are constructed to seek evidence of the development of human potential, of psychological functioning. As Waterman et al. (2010), p. 5 stated: “The objective elements include those behaviors involved in the pursuit of eudaimonic goals such as self-realization entailing the identification and development of personal potentials and their utilization in ways that give purpose and meaning to life.” Indicators of flourishing aim to demonstrate the development of human potential regarding being (character and virtue, being a good person, personal growth), having (a purpose in life, meaning in life, values, goals, positive relations with others, supportive relationships, close social relationships, self-acceptance, autonomy, environmental mastery, mastery accomplishment, optimism, engagement, health), and doing (the pursuit of excellence and self-realization, self-discovery, perceived development of one’s best potentials). This more comprehensive view of the human being, objective and generalizable enough to encompass people of different conditions and cultures, could serve as a reference framework for research on assessment and therapeutic intervention. The shift from the general to the specific, including individuals and cultural contexts, constitutes a research and intervention pathway. This approach has been partially initiated through proposals such as those by Bauer and Weatherbie (2023) and Fowers et al. (2024), which aim to enhance flourishing assessment tools by incorporating diverse cultural perspectives. Additionally, it is reflected in the emerging new directions in clinical therapy (Freetly Porter et al., 2023) and educational counseling (Weziak-Bialowolska et al., 2021).

Arnold, through Thomas Aquinas, is in contact with the notion of Aristotelian happiness-eudaimonia. Eudaimonia, as an idea, has been closely related since its origins to emotional regulation. For Aristotle, ethical virtue is a *sine qua non* condition of authentic happiness. As is known, in Aristotle, ethical virtues are strengths of character (ethos in Greek means character), among which are some (such as temperance and fortitude) whose function is to regulate emotions. Thus, emotional regulation is conceived by the eudaimonistic ethical tradition as a condition of eudaimonia-happiness. If we approach the concepts of flourishing and eudaimonia, therefore, we have here the historical origins of the connection between emotional regulation and flourishing. With Arnold’s conception of emotions strongly rooted in that same tradition, it is natural that he has concerned himself with studying the relationship between happiness and emotional regulation, in which the concept of self-ideal also plays a fundamental role.

After demonstrating the connection between Arnold’s self-ideal and the current conception of flourishing, we conclude that flourishing should be a primary goal in emotional regulation and that it should be reflected in the strategies employed to regulate emotion. In addition, we have also verified that theories on flourishing include references to emotions and their regulation. Vittersø (2016b), in his study of the role of emotions in relation to flourishing, acknowledges Arnold’s theory regarding the role of feelings and explains the importance of emotions in consolidating psychological functioning and being able to achieve the goals that individuals set in life. However, what we propose is that Arnold theorizes about emotions and the self-ideal not only to establish that emotional regulation is a means to reach the self-ideal, but also to suggest that the presence of a self-ideal serves emotional regulation, understanding by self-ideal not only a realization of one’s own potentialities but also achieving a good life. This idea relates to ongoing research. For example, some studies propose models of strategies that integrate the process of self-control with emotional regulation (Werner and Ford, 2023). Self-control focuses on regulating behaviors, while emotional regulation aims to regulate emotions. All cases of self-control include emotional regulation, but not all emotional regulation processes can be considered self-control. The goals for which behaviors are chosen could be oriented toward flourishing, and valuing them could be a strategy for emotional regulation.

Based on the debate raised by Hervás and Jódar (2008) questioning what marks the difference between a functional strategy and one that is not, we suggest that precisely the concept of self-ideal should be the direction in which emotion is regulated. In this sense, it is crucial to reflect on Arnold’s considerations about the motivating role of emotion, as well as the presence of motivation that can be constructive or not. Tamir et al. (2020) also highlight that emotional regulation is a motivated process, a matter that has often been relegated to the background in research. In this line, Tamir and Ford (2009) indicate that research in emotional regulation has assumed that individuals seek to feel good and avoid feeling bad, but often this is not the case, and individuals “may be motivated to experience even unpleasant emotions when they might be useful for goal attainment” (p. 488). This research corroborates what Arnold et al. (1962) pointed out: not only emotions matter but also the motives that drive my actions.

For emotional regulation to reflect flourishing, it is necessary to include eudaimonic motives, as referred to by Tamir (2016), that is, a sense when carrying out emotional regulation that goes beyond adaptation. Following Arnold et al. (1962), it is very relevant to know whether the individual is driven by constructive motivation toward an appropriate self-ideal or, in more current terms, flourishing. In this line, Roth et al. (2019) propose an approach to emotional regulation linked to aspects related to the self-ideal and flourishing (self-regulated action, short-and long-term goals, values, and preferences).

On the other hand, we can also conclude that emotions can be helpful in achieving flourishing if they are in harmony with the self-ideal; otherwise, it will require the use of different emotional regulation strategies to modulate their intensity and continue moving toward flourishing. Arnold suggests various strategies for modulating the intensity of emotions: the use of imagination, reflective reappraisal, corrective experiences, among others. This coincides with and expands current research showing that flourishers prioritize cognitive reappraisal as an emotional regulation strategy. Some authors (Hervás, 2011; Roth et al., 2019) suggest the need to include acceptance and non-judgmental embrace of emotional experience. At this point, it would be necessary to continue investigating appropriate emotional regulation strategies to achieve flourishing, based on Hervás (2011) emotional processing model, which, in turn, coincides in various aspects with Arnold’s proposal. Specifically, when considering conducting an emotional analysis to decide whether the information provided by the emotion is a “false alarm” or not, which in Arnold’s words would coincide with the affective memory that generates a disproportionate reaction to the situation.

Emotional regulation and flourishing are, therefore, two concepts that need each other, with a need to focus research on the interdependence between them. There is no flourishing without the regulation of our emotions; emotional regulation does not reach its deepest meaning if it is not directed toward flourishing. This holistic approach offers a comprehensive framework by integrating both realities, introducing an innovative perspective to the most recent research, which has been primarily focused on a more analytical view of each field separately.

We highlight two limitations of this study. The first limitation is the inability to use Magda Arnold’s unpublished writings, to which we had access, and which illustrate the developed argument in greater depth. The second limitation is the need to exclude other concepts and topics related to the central subject of this study, as well as the difficulty in delving deeper into the comparative analysis between different terms that share conceptual aspects. This last limitation also presents an opportunity for future research, such as examining the processes of emotional dysregulation. For instance, the third volume of Arnold’s unpublished masterpiece, Emotion and Personality, which addresses emotional disorders, could be used for this purpose. It could be studied the relationship between emotional dysregulation and flourishing, answering the question of why and how emotional dysregulation constitutes a difficulty or problem for flourishing. In addition, research could be conducted to examine the relationships between the following variables that highlight a problem of great relevance today: emotional dysregulation, personality traits, internet addiction and flourishing.

The conclusions reached in this article call for a review of intervention programs related to emotional regulation. Given the close relationship between emotional regulation and flourishing, such programs should address not only the identification and regulation of emotions but also elements such as purpose, meaning, and close, positive relationships–key aspects typically assessed by flourishing instruments. Therefore, future research could focus on identifying indicators that jointly evaluate emotional regulation and flourishing. Moreover, Hervás’s emotional processing model could be further explored and integrated with the concept of flourishing in his proposal; studies could also focus on emotional regulation strategies and use flourishing as a guide to determine whether the employed strategies are appropriate or not.

Finally, future research lines could propose the integrated framework outlined in this article as a basis for models of emotional and character education, and even for establishing new psychotherapy protocols.

## Author contributions

FR-F: Writing – original draft, Writing – review & editing. AB-M: Writing – original draft, Writing – review & editing. ME: Writing – original draft, Writing – review & editing.
